# A Case of Fulminant Amebiasis in a Female Commercial Sex Worker in Japan Requiring Subtotal Colectomy: A Literature Review and Case Report

**DOI:** 10.70352/scrj.cr.25-0434

**Published:** 2025-12-24

**Authors:** Seigo Kubota, Fuminori Mihara, Mai Nakamura, Takashi Kokudo, Yuichiro Mihara, Fuyuki Inagaki, Norihiro Kokudo

**Affiliations:** Hepato-Biliary-Pancreatic Surgery Division, Department of Surgery, National Center for Global Health and Medicine, Japan Institute for Health Security, Tokyo, Japan

**Keywords:** fulminant amebiasis, commercial sex worker, invasive amebiasis, *Entamoeba histolytica*, sexually transmitted infections, bowel perforation, emergency surgery

## Abstract

**INTRODUCTION:**

Fulminant amebiasis is a rare, potentially lethal condition caused by *Entamoeba histolytica*. It causes intestinal perforation and generalized peritonitis, and treatment requires an emergency operation and the administration of anti-amoebic drugs. Although *E. histolytica* infections are more commonly reported in men who have sex with men, we report a successfully treated case of fulminant amebiasis in a female commercial sex worker (CSW). This report presents a review of previously reported cases.

**CASE PRESENTATION:**

A 41-year-old woman with a 4-year history as a CSW presented to the emergency department with abdominal pain and diarrhea. She was admitted with acute colitis of unknown etiology. Stool tests performed after admission revealed *E. histolytica*, confirming the diagnosis of amoebic colitis, and treatment with metronidazole was initiated. On day 8 of treatment, the patient’s respiratory status worsened. Abdominal CT revealed a perforation of the transverse colon, leading to the diagnosis of fulminant amebiasis. Emergency laparotomy including subtotal colectomy and ileostomy was performed. Postoperatively, the patient’s condition gradually improved without complications, and she was discharged on POD 97 after finding housing. We reviewed and analyzed 52 reported cases of fulminant amebiasis, including the present case, treated with bowel resection in Japan since 2000. According to our literature review, the mortality rate was 28.8% and only 9.6% of patients were diagnosed with amebiasis at the time of hospital admission. Gastrointestinal perforation or necrosis requiring surgical intervention occurred after admission in 72.9% of patients. The mortality rate was 23.0% in patients who received anti-amoebic agents preoperatively compared to 30.7% in those who did not. Notably, the mortality rate reached 100% for patients where anti-amoebic agents were not administered, suggesting that such treatment is essential for survival (p = 0.005).

**CONCLUSIONS:**

Here, we present a successfully treated case of fulminant amebiasis in a female CSW. Analysis of previously reported cases suggests that the early administration of anti-amoebic agents is important for survival.

## Abbreviations

CSW
: commercial sex worker
HIV
: human immunodeficiency virus
MSM
: men who have sex with men

## INTRODUCTION

Invasive amoebiasis is a parasitic infection caused by *Entamoeba histolytica*. The infection is transmitted via the fecal–oral route through cysts present in the feces of infected individuals. Approximately 1000 cases are reported annually in Japan.^1)^ Although it was previously regarded as an imported infection from developing countries, the number of domestic cases has increased in recent years, accounting for over 80% of all cases. More than 80% of infected individuals are male, and male-to-male sexual contact is the primary route of transmission. However, infections among female CSWs have also increased, suggesting expansion as sexually transmitted infection.^2–6)^

Approximately 10% of individuals exposed to *E. histolytica* develop invasive amebiasis after a few weeks of incubation, while 90% become asymptomatic carriers. The asymptomatic infection usually lasts approximately a year,^7)^ with 20% of patients progressing to invasive amebiasis and the remaining 80% resolving spontaneously.^8)^

The main clinical forms of invasive amebiasis include amebic colitis, amebic liver abscess, and fulminant amebiasis. Amebic colitis typically presents with symptoms such as diarrhea, bloody stools, abdominal pain, and fever. In severe cases, ulcerative lesions can extend throughout the colon. These lesions often resemble ulcerative colitis on endoscopy and require careful differentiation.

Fulminant amebiasis is characterized by the progression of ulcerative lesions, leading to colonic perforation and generalized peritonitis, which is a life-threatening condition.^9)^ Emergency bowel resection surgery is essential for survival,^10)^ whereas postoperative administration of anti-amebic drugs such as metronidazole (MNZ) is critical to prevent recurrent perforations and ensure a complete cure.

Here, we present a case of fulminant amebiasis in a female CSW, along with a review of previously reported cases.

## CASE PRESENTATION

A 41-year-old female presented with abdominal pain. She had untreated diabetes mellitus. She had no family history and had not traveled overseas. During the admission interview, it was revealed that she was a CSW with a 4-year work history. Her last sexual intercourse was 8 months prior to presentation. Although she had not engaged in anal intercourse, she reported engaging in oral sex. Four days before the presentation, she developed abdominal pain and diarrhea, which progressed to difficulty eating. As her condition worsened, her mobility became increasingly impaired, necessitating transportation to our hospital. This was her first visit to our hospital.

Upon admission, physical examination revealed that the patient was 163 cm tall and weighed 75 kg, with a body mass index of 28.2. Vital signs included a blood pressure of 81/62 mmHg, a pulse of 106/min (regular), temperature of 34.6°C, and a SpO_2_ of 95% on room air. An abdominal examination revealed distension with rebound tenderness most pronounced in the upper abdomen.

The blood tests on admission were as follows: the white blood cell count was significantly elevated at 30940/μL, and the C-reactive protein level was markedly high at 36.09 mg/dL, indicating significant inflammation. Blood urea nitrogen was 29.3 mg/dL, and creatinine was 1.72 mg/dL, indicating renal impairment. The hemoglobin A1c level was elevated at 11.5%. The patient tested negative for HIV antibody, hepatitis B surface antigen, and rapid plasma reagin, but positive for hepatitis C virus and chlamydia antibodies (**Table 1**).

**Table 1:** Blood tests on admission

Hematology		Biochemistry	
WBC	30940/μL	TP	6.4 g/dL
RBC	5.83 × 10^6^/μL	Alb	1.9 g/dL
Hb	13 g/dL	T. Bil	0.3 mg/dL
Hct	40.5%	AST	23 U/L
Plt	71.1 × 10^4^	ALT	13 U/L
Neutro	89.3%	LDH	318 U/L
Lymph	8.1%	γ-GTP	22 U/L
Mono	1.9%	CK	33 U/L
Eosino	0.1%	BUN	29.3 mg/dL
Baso	0.6%	Cre	1.72 mg/dL
		Na	134 mEq/L
Infection marker		K	3.7 mEq/L
HIV Ag	(−)	Cl	88 mEq/L
HIV Ab	(−)	Glu	205 mg/dL
HBs Ag	(−)	HbA1c	11.5%
HCV Ab	(+)	CRP	36.09 mg/dL
RPR	(−)		
TPHA	(−)		
Chlamydia IgA	(+)		
Chlamydia IgG	(+)		

Alb, albumin; ALT, alanine aminotransferase; AST, aspartate aminotransferase; baso, basophils; BUN, blood urea nitrogen; CK, creatine kinase; Cre, creatinine; CRP, C-reactive protein; eosino, eosinophils; *γ*-GTP, gamma-glutamyl transferase; Glu, glucose; Hb, hemoglobin; HbA1c, hemoglobin A1c; HBs Ag, hepatitis B surface antigen; Hct, hematocrit; HCV Ab, hepatitis C virus antibody; HIV Ab, human immunodeficiency virus antibody; Ig, immunoglobulin; LDH, lactate dehydrogenase; lymph, lymphocytes; mono, monocytes; neutro, neutrophils; Plt, platelet; RBC, red blood cell; RPR, rapid plasma regain; T. Bil, total bilirubin; TP, total protein; TPHA, *Treponema pallidum* hemagglutination assay; WBC, white blood cell

**Fig. 1:**
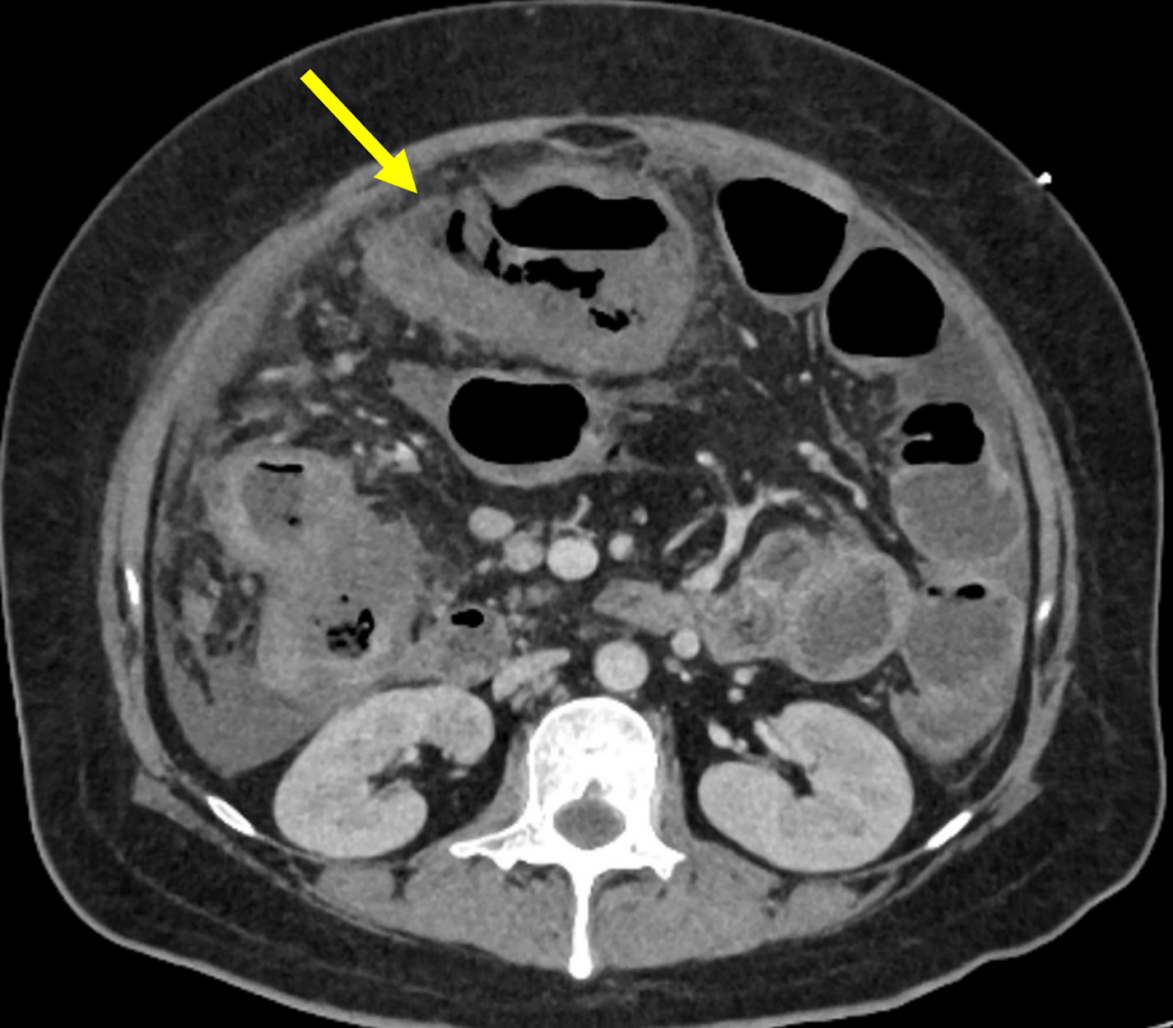
Abdominal CT on admission. The wall of the transverse colon thickened (yellow arrow), and the density of the surrounding fat tissue increased. A reduction in contrast enhancement was observed in parts of the transverse colonic wall.

Abdominal CT revealed thickening of the transverse colon wall and an increased density of the surrounding fat tissue. A reduction in contrast enhancement was observed in a part of the transverse colon wall (**Fig. 1**). CT also revealed good vascular enhancement up to the marginal artery. Free air, ascites, or liver abscesses were not detected.

After admission, the patient was diagnosed with acute colitis of unknown origin, paralytic ileus, and acute renal failure, and conservative treatment was planned. On the 2nd day of admission, consultation with our Infectious Disease Department led to a stool test using multiplex polymerase chain reaction (PCR), in which *E. histolytica* trophozoites were observed. MNZ 1500 mg/day and ceftriaxone 2 g/day were started intravenously on the same day. Due to severe dehydration caused by paralytic ileus and diarrhea, the patient received large volumes of fluid intravenously daily. Gradually, her overall condition improved. By day 4, intravascular dehydration improved, with a urine output of 1.1 mL/kg/h. In parallel, body weight gain and pleural effusion associated with fluid overload began to subside.

**Fig. 2:**
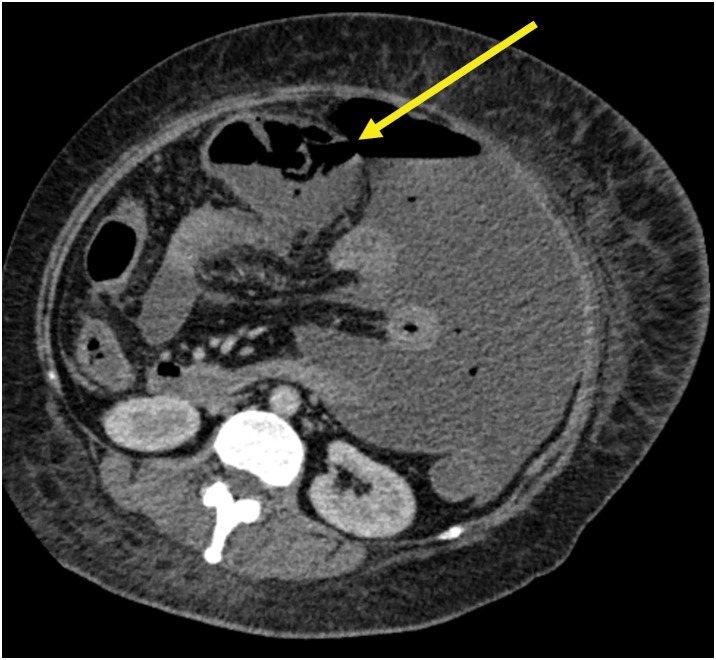
Abdominal CT on hospital day 8. Perforation of the transverse colon wall (yellow arrow), accompanied by massive ascites and free air, was observed.

She began drinking water on the 6th day of hospitalization and was transferred to the Infectious Disease Department on the 7th day. On day 8 of hospitalization, the patient experienced a sudden decline in oxygenation (SpO_2_, 92% on nasal high-flow oxygen at 50 L/min and 50% fraction of inspired oxygen), accompanied by tachypnea (respiratory rate, 24/min) and tachycardia (130 beats/min). Abdominal CT revealed massive ascites and free air with discontinuity in part of the transverse colon wall (**Fig. 2**). Acute generalized peritonitis and septic shock caused by transverse colon perforation were suspected, and an emergency operation was performed on the same day.

**Fig. 3:**
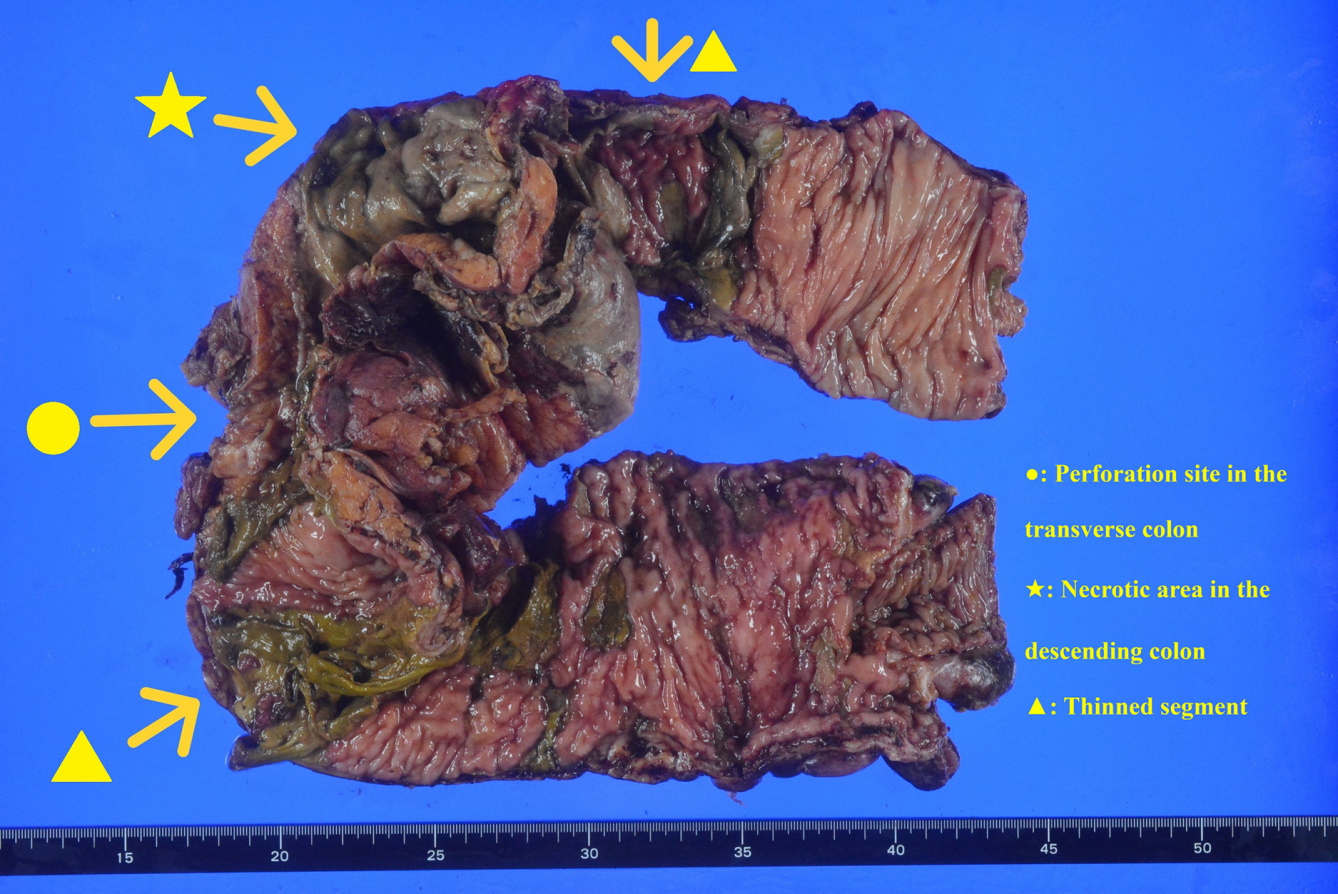
Resected specimen. The terminal ileum and descending colon were resected. A perforation was also observed in the transverse colon. From the transverse to the descending colon, the bowel wall appeared thinned and fragile, showing a “ragged rag-like appearance.”

Upon opening the abdomen, yellowish serous ascites was observed. A total of 4 L of ascitic fluid mixed with food residue, but without a fecal odor, was drained. Perforations are observed in the central transverse colon. The entire colon, particularly the cecum and descending colon, showed a mixture of thick and thin bowel walls. The descending colon wall was partially circumferentially necrotic, and lacerations occurred when it was mobilized from the retroperitoneum. The sigmoid colon and rectum showed no thinning suggestive of necrosis and appeared normal on gross inspection. Based on the presence of overt mucosal necrosis, the resection area was determined to include the transverse and descending colon. Subtotal colectomy was performed from the terminal ileum to the descending colon, and an ileostomy was performed. The mucosa of the remaining bowel was inspected through the cut end of the descending colon and was confirmed to be grossly normal. Intraoperative colonoscopy was not performed. The resected bowel wall was thinned, perforated, and necrotic with a rag-like appearance. Numerous scattered ulcerative lesions were observed. A perforation was observed in the transverse colon (**Fig. 3**).

Pathological findings revealed hemorrhage, inflammatory cell infiltration, and necrosis around the perforation site in the transverse colon; however, *E. histolytica* was not detected.

**Fig. 4:**
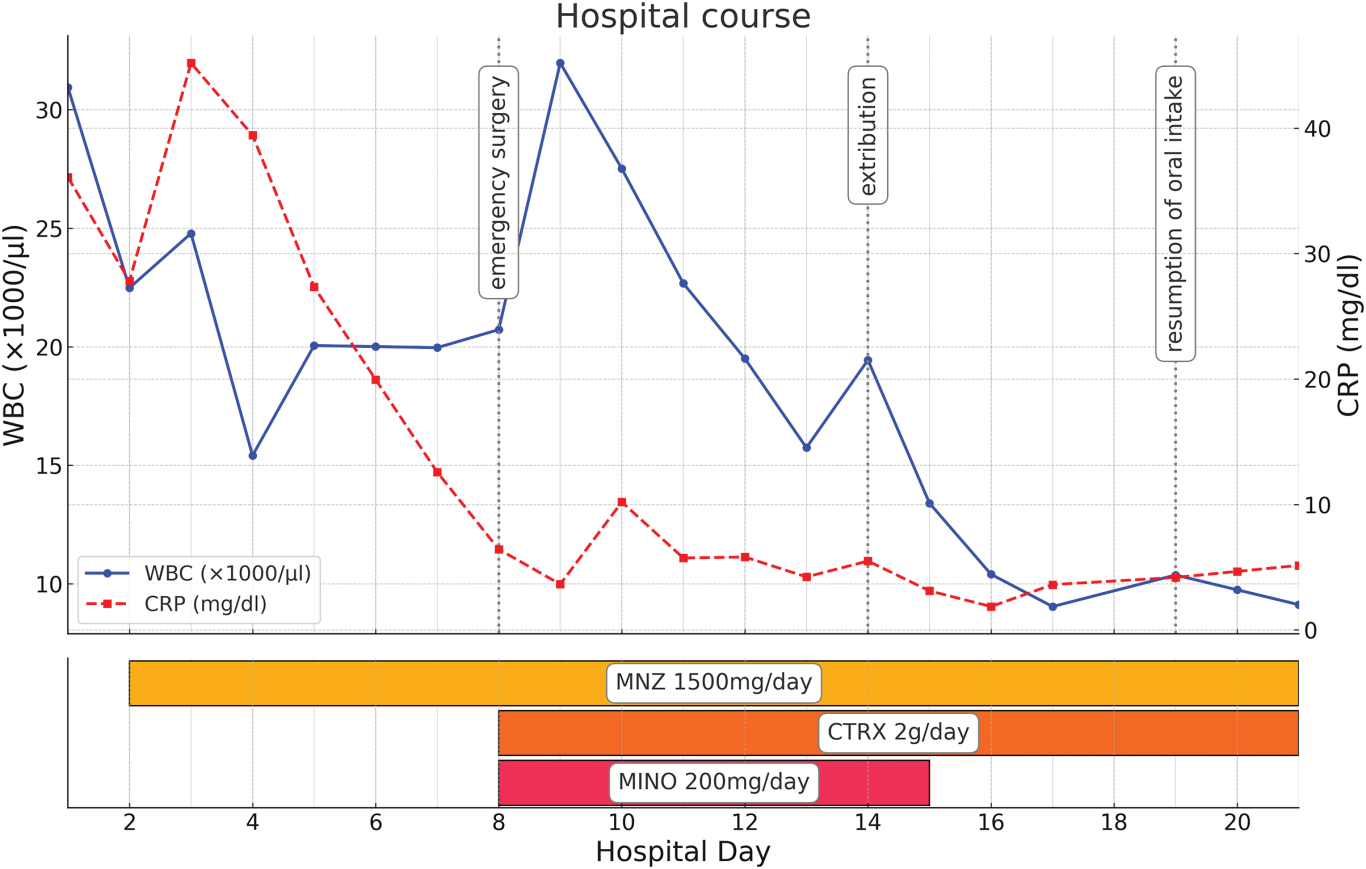
Hospital course. Emergency operation was performed on hospital day 8. Extubation was performed on day 14 of hospitalization. Oral intake resumed on day 19 of hospitalization. MINO was administered from hospital day 8 to 15. MNZ and ceftriaxone were administered from hospital day 8 to 21. CRP, C-reactive protein; CTRX, ceftriaxone; MINO, minocycline; MNZ, metronidazole; WBC, white blood cell

Postoperatively, treatment with MNZ 1500 mg/day was continued. The patient’s overall condition gradually improved, without complications (**Fig. 4**). She resumed eating on the 11th POD and was switched from MNZ to oral paromomycin 1500 mg/day for 10 days as a radical treatment for the remaining cyst on the 13th POD. Oral antibiotics were administered starting on the 32nd POD to treat an intra-abdominal abscess, which improved. Despite a generally good postoperative course, discharge was delayed to the 97th POD due to housing issues. An operation to close the ileostomy was performed on the 179th POD.

## DISCUSSION

In the present case, the diagnosis was made early during hospitalization through stool testing using multiplex PCR. Our hospital is located in Shinjuku Ward, which contains the largest entertainment district in Japan, and we frequently see patients who are CSWs or MSM. Between two and five cases of amebiasis are diagnosed at our institution each year. Although this test is not covered by the national health insurance system, it can be performed at the discretion of the infectious disease physician considering the patient’s background as a CSW. Routine stool testing is generally of limited utility in cases of enteritis; however, it may be useful in patients with high-risk backgrounds, such as MSM or CSWs. In Japan, direct smear microscopy, formalin–ether concentration techniques, and rapid stool antigen tests are covered by insurance. Although all of these tests have high specificity, the sensitivity of microscopy is reported to be 50%–80%, and that of rapid antigen testing is approximately 60%. It should also be noted that sensitivity may be further reduced when formed stool is used.^11,12)^

In this case, *E. histolytica* was not identified in the resected specimen. This suggests that short-term administration of anti-amoebic therapy was effective. However, despite apparent clinical improvement, bowel perforation occurred on day 6 of treatment. *E. histolytica* is known to cause mucosal ulceration by invading the submucosa and inducing apoptosis, in addition to its cytolytic activity. Furthermore, changes in intestinal permeability may occur independently of cytolytic activity, resulting in watery diarrhea.^13)^ In the present case, although tissue injury caused by *E. histolytica* may have been controlled by anti-amoebic therapy, irreversible bowel damage had likely already occurred. Consequently, the intestine may have failed to withstand the increased intraluminal pressure due to persistent watery diarrhea, leading to perforation. The healing of bowel injury may have been delayed due to untreated diabetes mellitus.

There is an ongoing debate regarding the optimal extent of bowel resection in surgery for fulminant amebiasis. Some reports suggest that only stoma creation should be performed,^14)^ whereas others recommend extensive colectomy.^15)^ In cases where anti-amoebic therapy has been administered preoperatively, as in the present case, the cause of perforation is thought to be delayed healing of bowel injury. Therefore, resection limited to the visibly damaged areas may be sufficient. Because tissue damage caused by *E. histolytica* originates from the mucosal surface, intraoperative colonoscopy can help identify affected areas more precisely and allow for a more accurate determination of the resection margins.

For the literature review, reports of bowel resection performed for fulminant amebiasis in Japan since 2000 were searched using the keywords “fulminant amebiasis” and “perforation” in Ichushi-Web and PubMed. Including our case, a total of 52 cases with available full-text articles were identified (**Table 2**). The chi-squared test, Student’s t-test, and Mann–Whitney U test were used as appropriate for statistical analysis (**Table 3**). The average age of the patients was 54.9 years, and the male-to-female ratio was 41:11 (78.8% male). The mortality rate was 28.8% (15 patients), which is notably higher than that of general gastrointestinal perforations.

**Table 2:** Summary of previously reported cases

Year	Author	Age	Sex	Transmission route	Underlying comorbidities	Initial diagnosis	Days*	Surgical procedure	Reoperation	Rag-like appearance	Days**	Anti-amebic drugs	Outcome
2000	Moriyama^24)^	61	M	Travel history^(a)^		Amebic colitis	19	Right hemicolectomy, ileostomy	No	–	1	Preoperative	Death
2002	Shimada et al.^25)^	62	M	Travel history (China)		Periappendiceal abscess	1	①Cecal resection	Yes	−	13	Postoperative	Death
								②Total colectomy, ileostomy					
2003	Hirakawa et al.^26)^	53	M	Sexual intercourse		Ileus	2	Total colectomy, ileostomy	No	–		None	Death
2006	Arai et al.^27)^	49	M		HIV	Bowel necrosis	1	Sigmoidectomy, ileocecal resection, ileostomy	No	+	1	Postoperative	Death
2007	Ueda et al.^28)^	77	M			Acute enteritis	3	①Subtotal colectomy	Yes	−		None	Death
								②Abdominoperineal resection					
2008	Kato et al.^29)^	50	M			Ileus	2	Total colectomy, ileostomy	No	+	7	Postoperative	Death
2009	Hanaoka et al.^30)^	52	M		Chemotherapy	Gastrointestinal bleeding	3	Colectomy	No	–	–	Postoperative	Death
2011	Kojo et al.^31)^	53	M	Travel history^(a)^, sexual intercourse	Diabetes	Acute appendicitis	2	①Abscess drainage	Yes	+		None	Death
								②Abscess drainage					
								③Cecal resection					
2012	Ohta et al.^32)^	66	F		Diabetes	Unknown	4	Total colectomy, ileostomy	No	+	7	Postoperative	Death
2015	Mori et al.^18)^	46	M		Hepatitis B-related cirrhosis	Spontaneous bacterial peritonitis	9	Right hemicolectomy, ileostomy	No	–	8	Preoperative	Death
2015	Kojima et al.^33)^	71	M	Travel history (Africa)	Chemotherapy	Infectious enteritis	27	Colectomy, ileostomy	No	+	26	Preoperative	Death
2017	Kinoshita et al.^34)^	76	M		HIV	Bowel perforation	1	Rectal resection, colostomy	No	–	1	Postoperative	Death
2018	Umemoto et al.^35)^	71	M		Cancer	Unknown	8	①Right hemicolectomy	Yes	−	16	Postoperative	Death
								②Total colectomy, small bowel resection, ileostomy					
2021	Tomino et al.^36)^	64	M		Cancer	Intestinal tumor	1	Subtotal colectomy, small bowel resection	No	–		None	Death
2023	Iwasaki et al.^37)^	72	M	Travel history^(a)^	Diabetes	Bowel necrosis	1	①Total colectomy	Yes	−	5	Postoperative	Death
								②Ileostomy					
2000	Takahashi et al.^38)^	61	M			Pericecal abscess	5	Subtotal colectomy	No	–	–	Postoperative	Survival
2003	Ishii et al.^39)^	31	M	Travel history^(a)^		Infectious enteritis	5	Subtotal colectomy	No	–	8	Postoperative	Survival
2003	Ishida et al.^40)^	48	M		Ulcerative colitis (steroid use)	Ulcerative colitis	7	Total colectomy, ileostomy	No	+	7	Postoperative	Survival
2003	Yukawa et al.^41)^	60	F		MTX, steroid use	Inflammatory bowel disease	16	Ileocecal resection	No	–	–	Postoperative	Survival
2004	Ohe et al.^15)^	33	M		Hepatitis B	Acute appendicitis	2	①Appendectomy, abscess drainage	Yes	+	−	Postoperative	Survival
								②Abscess drainage					
								③Ileocecal resection, ileostomy					
								④Total colectomy, ileostomy					
2004	Mizutani et al.^14)^	72	M			Ulcerative colitis	6	Sigmoidectomy, colostomy	No	+	6	Postoperative	Survival
2006	Miyahara et al.^42)^	25	M			Liver abscess	5	①Abscess drainage	Yes	−	−	Postoperative	Survival
								②Partial hepatectomy					
								③Sigmoidectomy, colostomy					
2007	Nakahira et al.^43)^	78	M	Travel history (China)		Perianal abscess, diverticulitis	3	Ileocecal resection, left hemicolectomy, colostomy	No	–	3	Postoperative	Survival
2008	Ishiyama et al.^44)^	37	M	MSM	HIV	Amebic colitis	2	Ileostomy	No	–	–	Preoperative	Survival
2008	Taketani et al.^45)^	54	M			Liver abscess	5	Right hemicolectomy, ileostomy	No	–	6	Postoperative	Survival
2008	Muto et al.^46)^	72	M	Travel history (USA)		Rectal cancer	10	Left hemicolectomy, abdominoperineal resection, colostomy	No	–	11	Postoperative	Survival
2008	Suzumura et al.^47)^	55	M	MSM	HIV, diabetes	HIV	35	Abdominoperineal resection	No	–	42	Postoperative	Survival
2009	Kosaka et al.^48)^	52	F			Diverticulitis	7	①Right hemicolectomy	Yes	–	24	Postoperative	Survival
								②Transverse colectomy, left hemicolectomy, colostomy					
2009	Hayami et al.^10)^	51	M		Diabetes	Bowel perforation	1	Right hemicolectomy	No	+	13	Postoperative	Survival
2010	Toriguchi et al.^49)^	70	M		PH of amebiasis	Unknown	2	①Right hemicolectomy, ileostomy	Yes	+	12	Postoperative	Survival
								②Total colectomy, small bowel resection, ileostomy					
2010	Saida et al.^50)^	55	M	Travel history^(a)^	Ulcerative colitis (steroid use)	Ulcerative colitis	48	Subtotal colectomy, ileostomy	No	+	42	Preoperative	Survival
2010	Endo et al.^51)^	44	M	MSM	HIV	Amebic colitis	1	①Hartmann’s procedure	Yes	+	6	Postoperative	Survival
								②Subtotal colectomy, ileostomy					
2011	Ishioka et al.^52)^	39	M	MSM, travel history^(a)^	HIV	Unknown	5	Right hemicolectomy	No	–	1	Preoperative	Survival
2011	Harata and Terashita^53)^	69	F		Diabetes, steroid use	Diverticulitis	—	①Right hemicolectomy	Yes	−	−	Postoperative	Survival
								②Ileostomy					
2011	Tamaki et al.^54)^	61	M	Travel history (China, USA)	Chemotherapy	Amebic colitis	—	Rectal resection, colostomy	No	–	–	Preoperative	Survival
2012	Kitamura et al.^55)^	60	F		Diabetes	Bowel perforation	1	①Sigmoidectomy, colostomy	Yes	−	9	Postoperative	Survival
								②Left hemicolectomy, colostomy					
2015	Mori et al.^18)^	21	F	Travel history^(a)^		Acute enteritis	5	Right hemicolectomy, ileostomy	No	–	–	Postoperative	Survival
2015	Mori et al.^18)^	49	M	MSM	HIV, hepatitis B	Amebic colitis	56	Total colectomy, small bowel resection, ileostomy^(b)^	No	+	–	Preoperative	Survival
2015	Goto et al.^56)^	30	F		Perinatal period	Bowel perforation	1	①Subtotal colectomy	Yes	−	15	Postoperative	Survival
								②Ileostomy					
2015	Takenoya et al.^57)^	53	F			Acute appendicitis	1	①Appendectomy, abscess drainage	Yes	−	62	Postoperative	Survival
								②Drainage					
								③Sigmoidectomy, ileocecal resection					
								④Drainage, debridement					
								⑤Perforation repair, debridement					
2016	Kubo et al.^58)^	65	M		Diabetes	Malignant lymphoma	2	Ileocecal resection	No	+	9	Postoperative	Survival
2017	Hasegawa et al.^59)^	57	M	Travel history^(a)^		Acute enteritis	5	Subtotal colectomy, ileostomy	No	–	8	Postoperative	Survival
2017	Ueno et al.^60)^	80	M			Unknown	6	Total colectomy, ileostomy	No	+	12	Postoperative	Survival
2017	Dei et al.^61)^	46	M		Diabetes	Acute appendicitis	1	Right hemicolectomy	No	–	9	Postoperative	Survival
2018	Tanaka et al.^62)^	40	M			Ischemic colitis	19	Subtotal colectomy, ileostomy	No	–	–	Postoperative	Survival
2021	Sugiyama et al.^63)^	48	F			Acute appendicitis	13	Small bowel resection, cecal resection, abscess drainage	No	–	20	Postoperative	Survival
2021	Kitaoka et al.^64)^	64	M	Travel history^(a)^	Chemotherapy	Intussusception	25	Colectomy, ileostomy	No	–	33	Postoperative	Survival
2021	Kato et al.^65)^	70	M	Travel history^(a)^	Diabetes Acute hepatitis A (steroid pulse)	Acute hepatitis	26	Right hemicolectomy, ileostomy	No	–	14	Preoperative	Survival
2022	Yasumura^19)^	50	M	MSM	Diabetes	Bowel perforation	75	Right hemicolectomy^(c)^	No	–	4	Preoperative	Survival
2023	Azuma et al.^20)^	27	F	Travel history^(a)^	Perinatal period	Acute enteritis	84	Subtotal colectomy	No	–	3	Preoperative	Survival
2023	Takiguchi et al.^66)^	65	M		Chemotherapy	Acute enteritis	26	Total colectomy, ileostomy	No	–	22	Preoperative	Survival
2025	Kubota (this study)	41	F	CSW	Diabetes	Acute enteritis	8	Subtotal colectomy, ileostomy	No	+	2	Preoperative	Survival

Reports of bowel resection performed for fulminant amebiasis in Japan since 2000 were searched using the keywords “fulminant amebiasis” and “perforation” in Ichushi-Web and PubMed.

*Days from admission to 1st operation.

**Days from admission to diagnosis or anti-amebic therapy.

^(a)^History of travel to Southeast Asia.

^(b)^Operation for toxic megacolon.

^(c)^Operation for chronic bowel stenosis.

CSW, commercial sex worker; F, female; HIV, human immunodeficiency virus; M, male; MSM, men who have sex with men; MTX, methotrexate; PH, potential of hydrogen

**Table 3:** Prognostic analysis of previously reported cases

	N (%)			p-Value
	Overall	Survival	Death	
No.	52	37	15	
Age (years), mean (SD)	54.9 (14.5)	52.2 (15.2)	61.5 (10.4)	0.036
Sex				
Male	41	27 (65.9)	14 (34.1)	0.145
Female	11	10 (90.1)	1 (9.1)	
Days from admission to 1st procedure, median [IQR]		5 [2.0, 17.5]	2 [1.0, 8.0]	0.112
Same day of admission	10	6	4	
Within 7 days	21	16	5	
More than 8 days	17	13	4	
Reoperation				
Yes	14	9 (64.2)	5 (35.7)	0.511
No	38	28 (73.6)	10 (26.3)	
Anti-amebic drugs				
Yes	48	37 (77.0)	11 (22.9)	0.005
No	4	0 (0.0)	4 (100.0)	
Preoperative anti-amebic drugs				
Yes	13	10 (76.9)	3 (23.0)	0.733
No	39	27 (69.2)	12 (30.7)	
Days from admission*, mean (SD)				
To first procedure		9.5 (11.2)	5.6 (7.6)	0.232
To diagnosis or anti-amebic drugs		15.8 (14.7)	8.5 (7.9)	0.147

The chi-squared test, Student’s t-test, and Mann–Whitney U test were used as appropriate for statistical analysis.

*Three elective surgical cases for chronic intestinal stricture or toxic megacolon^19,22,23)^ were excluded.

IQR, interquartile range; SD, standard deviation

In terms of transmission route, sexual contact was considered the likely cause in 9 cases. The patients in 16 cases had a history of overseas travel, including 11 who had traveled to Southeast Asia. In 29 cases, the transmission route was unknown, and some patients had no history of overseas travel. No cases involving CSWs were identified except for our case. Additionally, our patient was a CSW without a history of anal intercourse, suggesting the possibility of transmission through oral–anal sexual contact.^2)^

Underlying conditions that increase the risk of developing fulminant amebiasis include steroid use, HIV infection, diabetes mellitus, alcohol dependence, malignancy/chemotherapy, and pregnancy, as reported in previous studies.^16,17)^ Among the 52 cases reviewed, diabetes mellitus was the most common comorbidity, found in 12 patients (22.6%), including the patient in our case. This was followed by malignancy/chemotherapy in seven cases, HIV in seven cases, steroid use in five cases, and pregnancy in two cases. In total, 30 of the 52 patients (57.6%) had one or more of these risk factors. Of these, 9 patients died and 21 survived. These risk factors may not only contribute to immunosuppression and increased disease severity but also delay mucosal healing of necrotic bowel tissue due to impaired wound healing.

Only 5 patients (9.6%) were diagnosed with amebiasis at the time of admission, indicating the difficulty of early diagnosis. Other initial diagnoses included acute enteritis or infectious enteritis in 7 patients, bowel perforation or necrosis in 7 patients, acute appendicitis in 5 patients, ulcerative colitis in 4 patients, and diverticulitis in 4 patients.

Emergency surgery was performed on the day of admission in 10 (19.2%) patients. The initial procedure was performed within 7 days of admission in 21 patients (40.3%), while 17 patients (32.6%) underwent their 1st procedure 8 days or more after admission. The average time from admission to operation was 15.5 days (median, 6.5 days). For most patients, gastrointestinal perforation or necrosis developed during admission, requiring emergency operations. Similarly, in our patient, gastrointestinal perforation occurred on the 8th day after admission and led to an emergency operation. The clinical course, in which gastrointestinal perforation or necrosis occurs after admission, is characteristic of fulminant amebiasis.

The mortality rate was 23.0% for patients who received preoperative anti-amoebic therapy compared to 30.7% for those who did not, suggesting a trend toward improved outcomes with the early administration of anti-amoebic drugs. Furthermore, all 4 patients in whom anti-amoebic drugs were not administered resulted in death. Administration of anti-amoebic agents was associated with a statistically significant improvement in the survival rate (p = 0.005), suggesting that their use is essential for patient survival. Including the patient in our case, 5 patients developed bowel perforation and required emergency surgery despite receiving anti-amoebic therapy for 3 days or more. Of these five cases, the patients in four cases had underlying risk factors for fulminant amebiasis, including diabetes mellitus, malignancy/chemotherapy, HIV infection, and steroid use. The remaining case involved severe hepatic and renal dysfunction. These findings suggest that even after initiating anti-amoebic therapy, bowel perforation can still occur in patients with risk factors for fulminant progression or in those with poor general condition. Therefore, careful monitoring is required in such cases.

Reoperation, including additional bowel resection or stoma creation, was required in 14 patients (26.9%). The mortality rate in patients who required reoperation was 35.7%, indicating a higher mortality rate. These patients had delayed diagnosis, leading to re-perforation or abscess formation after the initial procedure. Without anti-amoebic treatment, bowel perforation may recur, often resulting in the need for reoperation. In contrast, no reoperation was required in patients who had received preoperative anti-amoebic therapy, suggesting that no new intestinal injury caused by *E. histolytica* had occurred. In our case as well, preoperative administration of anti-amoebic drugs was effective. *E. histolytica* was not detected in the resected specimen, and no recurrence of perforation occurred.

We also analyzed the number of days from admission to diagnosis and from admission to the initial surgery. Three elective surgical cases for chronic intestinal stricture or toxic megacolon^18–20)^ were excluded. Both these intervals tended to be longer in the survival group than in the fatal group; however, the differences were not significant. On average, it took more than 3 days after surgery to establish the diagnosis. Because the severity of illness at admission varied across cases, simple comparisons based on days should be interpreted with caution. Although early diagnosis is thought to improve survival, it is often difficult to achieve in practice. In many cases, the diagnosis is made based on pathological findings from the resected specimen. Developing strategies for earlier diagnosis remains a key challenge.

An intraoperative finding suggestive of fulminant amebiasis is a “ragged rag-like appearance” or “wet blotting paper-like appearance,” which refers to extreme fragility of the bowel wall. This finding describes a condition in which the intestinal wall becomes severely thinned due to necrosis and liquefaction, to the extent that even gentle traction during surgery can cause perforation.^18,21,22)^ Among the 52 reviewed cases, this appearance was explicitly documented as an intraoperative or pathological finding in 16 cases (30.7%), including our case. Thus, the ragged rag-like appearance is an important finding that may assist in the intraoperative diagnosis of fulminant amebiasis and should be recognized by surgeons.

This study has some limitations. First, fulminant amebiasis itself may be significantly underreported in the literature. Second, as this is a retrospective study based on previously published case reports, the data collected were incomplete due to limitations in the original case descriptions. Finally, the small sample size may have limited the statistical power to detect significant associations.

This case represents the first case report in Japan of fulminant amebiasis requiring bowel resection in a female CSW within the scope of our search. The key factors contributing to the successful outcome in our case included early diagnosis after admission, initiation of anti-amoebic drug therapy preoperatively, and the ability to avoid reoperation during the acute phase.

With the increasing spread of sexually transmitted infections in Japan, as exemplified by the recent syphilis outbreak, there is growing concern regarding the spread of amebiasis. In a voluntary testing and counseling center in Tokyo that offers free HIV and syphilis testing, the seropositivity rate for *E. histolytica* antibodies was 2.64%, surpassing the HIV positivity rate of 0.34% and the syphilis rapid plasma reagin antibody positivity rate of 2.11%.^23)^ HIV-positive individuals and MSM have traditionally been considered high-risk groups for amebiasis.^5,6)^ However, the present case highlights the importance of also considering amebiasis in patients with a background as a CSW, as early suspicion can lead to timely diagnosis and treatment.

## CONCLUSIONS

Here, we report the successful treatment of fulminant amebiasis in a female CSW. Previous case reports suggest that early preoperative diagnosis and administration of anti-amoebic drugs are critical for survival.

## DECLARATIONS

### Availability of data and materials

The datasets supporting the conclusions of this article are included within the article and its additional files.
